# Distribution Analysis of *Candida albicans* according to Sex and Age in Clinical Specimen Testing for Sexually Transmitted Diseases

**DOI:** 10.4014/jmb.2208.08029

**Published:** 2022-11-03

**Authors:** Jae Eun Choi, Jae-Sik Jeon, Jae Kyung Kim

**Affiliations:** 1 Department of Public Health Science, Dankook University Graduate School, Chungnam 31116, Republic of Korea; 2 Department of Biomedical Laboratory Science, Dankook University College of Health Sciences, Chungnam 31116, Republic of Korea

**Keywords:** *Candida albicans*, real-time polymerase chain reaction, sexually transmitted disease, mycology

## Abstract

The prevalence of candidiasis, a contagious disease with high morbidity and mortality, has sharply increased globally over the last two decades. *Candida albicans* can cause serious infections in patients with weak immunity and in recipients of prolonged antibiotic treatment. Consequently, rapid and accurate identification of species can play an important role in the treatment of candidiasis. Here, we investigated the positive rate and infection trend of *C. albicans* according to age, specimen type, and sex using multiplex real-time polymerase chain reaction-based testing of samples collected for the diagnosis of sexually transmitted diseases in Korea between 2018 and 2020. When the type of specimen collected was a swab, the positive rate of *C. albicans* was higher among younger women, and tended to decrease with age. Analysis of swab samples revealed higher positive rates than urinalysis. The reduction trend in positive rates by age was comparable between the overall samples and urine specimens. Among male patients, the positive rate did not differ substantially across the various types of specimens collected. Previous studies have shown a higher prevalence of non-albicans *Candida* species than *C. albicans* in clinical specimens, and exclusion of the former from our analysis may be a limitation of this study. However, our findings contribute significantly to the literature because globally, there is a paucity of epidemiological studies using molecular techniques to detect *C. albicans* in sexually transmitted disease test samples.

## Introduction

*Candida albicans* inhabits the human oropharyngeal and gastrointestinal tracts, vulvovaginal region, and skin. It causes localized surface mucosal skin conditions and various life-threatening invasive diseases [1], particularly in individuals with weak immunity and in recipients of prolonged antibiotic treatment [2, 3]. For example, balanitis or vaginitis can occur secondary to diabetes or other immunodeficiency syndromes [4]. Urinary tract infections caused by *Candida* spp. may present with severe symptoms, leading to candidemia and sepsis [5].

The prevalence of candidiasis, a contagious disease with high morbidity and mortality, has sharply increased globally over the last two decades [6, 7]. A gradual increase in resistance to antifungal drugs has been noted among non-albicans *Candida* spp. As a result, clinical treatment with antifungal agents has become challenging [8].

To date, various epidemiological studies on *Candida* infections have been conducted [9-11]. The currently available subtype classification methods include restriction fragment length polymorphism analysis, multilocus enzyme electrophoresis, and Southern blotting using repetitive DNA probes [9, 10]. Furthermore, molecular analytical methods, such as blot hybridization, random amplification of polymorphic DNA, nuclear type analysis (*i.e.*, electrophoretic karyotyping) using pulsed-field gel electrophoresis, and chromosomal DNA limiting enzyme analysis, have been attempted [11-14]. Mechanical analysis using polymerase chain reaction (PCR) has become quite common in recent years. Advances in these assays allow for the rapid identification of Candida strains, which may play an important role in the treatment of candidiasis.

In the current study, we aimed to determine *C. albicans* positive rates and infection trends, using real-time multiplex PCR (mPCR), in specimens collected at a clinical laboratory in South Korea from participants suspected of having sexually transmitted diseases (STDs).

## Materials and Methods

This study was approved by the Dankook University Institutional Review Board (IRB file No. DKU 2021-04-002) and was conducted in conformance with the tenets of the Declaration of Helsinki.

### Materials

From September 2018 to December 2020, specimens were collected (*n* = 59,381) from outpatients across primary and secondary hospitals in Korea. U2Bio (Korea) conducted molecular biological testing of the specimens for venereal diseases. The specimens were analyzed after classification into catheter, pus, tissue, swab, and urine sample groups.

### Nucleic Acid Extraction

Clinical specimens were stored at -70°C until required for DNA isolation for real-time mPCR. DNA for the mPCR assay was extracted using an ExiPrep Dx Bacteria Genomic DNA Kit (Bioneer, Korea) according to the manufacturer’s instructions.

Concentrations of the extracted DNA samples were measured using an AccuPower STI4C-Plex Real-Time PCR Kit (Bioneer). Undiluted DNA samples (>20 μg/ml) were quantified using a spectrophotometer (NanoDrop 1000; Thermo Fisher Scientific, USA).

### Real-Time PCR Analysis

Real-time PCR analysis was performed using the AccuPower STI4C-Plex Real-Time PCR Kit with an Exicycler 96 Real-Time Quantitative Thermal Block (Bioneer), according to the manufacturer’s instructions. The PCR mixture was prepared by adding 1 μl of IPC to 44 μl of DEPC DW per reaction; the mixture was vortexed and spun down for > 10 s. To compensate for any loss, at least one tube more than the required amount was calculated for. The amplification protocol consisted of one cycle at 95°C for 5 min, followed by 45 cycles at 95°C for 5 s, and 55°C for 5 s. The threshold cycle was determined based on the manufacturer’s instructions. Four pathogens, including *C. albicans*, *Gardnerella vaginalis*, *Ureaplasma parvum*, and *Treponema pallidum*, were analyzed; however, we reported only the *C. albicans* data in this study. The limit of detection of *C. albicans* using the AccuPower STI4C-Plex Real-Time PCR Kit is 147.9 copies/ml. Positive control DNA concentrations are 3.85 × 10^5^, 3.85 × 10^4^, and 3.85 × 10^3^ copies/ml.

### Statistical Analysis

SAS version 9.4 (SAS Institute Inc., USA) was used to perform all statistical analyses, including descriptive and frequency analyses. *C. albicans* DNA positive rates detected using real-time PCR were analyzed according to sex, age, and specimen type. *p* < 0.05 was considered significant.

## Results

Of the 59,381 collected specimens, 1,941 tested positive for *C. albicans*. Among those, 381 and 1,560 specimens were obtained from male and female patients, with positive rates of 0.8% and 11.5%, respectively (Table 1). Teenagers had the highest positive rate at 7.4%, followed by patients in their 40s (3.7%), 20s (3.5%), and 30s (3.2%), respectively. We combined the data on patients aged ≥ 70 years for further analysis, because the number of positive patients decreased with age (Table 1). Positivity according to sex was 0.7–1.6% for males, similar in all age groups, but 4.5–22.7% for females, showing a large difference. The average age at incidence was 38.3 ± 14.6 years, with 40.9± 14.0 and 37.6 ± 15.6 years for male and female patients, respectively.

Positive rates were highest at younger ages and tended to decrease with age (*p* < 0.05). The distribution patterns of positivity by age, for both men and women, in the overall sample were comparable to those observed in the urinalysis data (Table 1).

**Fig. 1:**
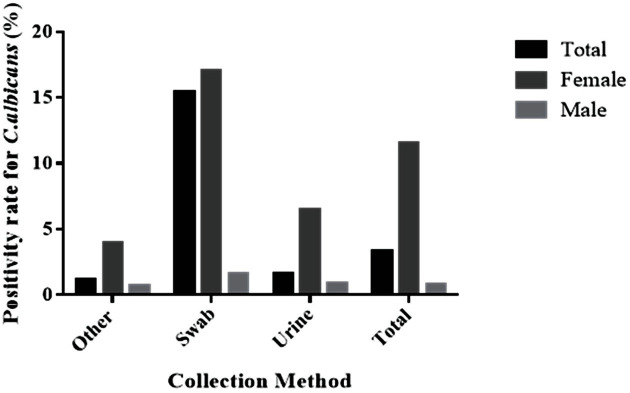
Comparison across the collected specimen types classified by sex. Positive rates of *C. albicans* corresponding to different sample acquisition methods used for total (black bar), male (light gray bar), and female (gray bar) patients.

According to the type of sample collected, swabs yielded the highest positive rate for *C. albicans* in males (15%) and females (17%) (Fig. 1).

## Discussion

*Candida* spp. are a part of the normal microbiota in humans; however, they may cause opportunistic infections. The molecular analysis of *Candida* spp. would be of great value in epidemiological studies and can help in the rapid identification of fungal pathogens, thereby reducing hospitalization and treatment costs associated with *Candida* infections [15]. We analyzed data collected for testing STDs and used mPCR to evaluate the correlation of age, sex, and type of specimen with *C. albicans* positivity in order to produce basic data for use in promoting public health.

We found that women aged < 40 years had a higher positive rate than those aged > 50 years. A study of swab-acquired specimens showed that women aged < 40 years were more than twice as likely to have vulvovaginal candidiasis (VVC) than older women, and that sexual intercourse first experienced under the age of 20 was associated with the risk of developing VVC [9]. Previous studies have shown that sexual intercourse increases the probability of developing VVC by up to four times [9]. PCR analysis of fungal isolates obtained from swabs revealed that 95/210 (45.2%) patients with vulvovaginitis were found to have VVC. The age range of women with VVC was 14–60 years, with most VVC cases observed in the 21–30-year age group [16]. Our results corroborated the findings of the above-mentioned studies and revealed that the highest *C. albicans* positive rate was seen among teenagers and those in their 20s, and the infection rates were high among women aged < 40 years, suggesting that sexual intercourse and positive rates of *Candida* spp. are correlated in women.

Analysis of urethral infections caused by *C. albicans* showed high female infection rates, similar to those reported previously [17]. The lower positive rates in men could be due to the possible antifungal properties of prostatic fluid [15, 18].

A previous study examined 650 specimens (vaginal, penile, urethral and ear swabs, as well as urine) collected from patients aged 20–69 years, and revealed that pregnant women (aged 20–43 years) had the highest proportion (73.3%) of *C. albicans* infections [19]. Furthermore, *C. albicans* was prevalent in 37.9% of women with urinary tract infections (UTIs), and of the 90 *Candida* spp. identified, *C. albicans* (45.5%) was isolated most frequently [19]. In the above-mentioned study, specimens were obtained from urine (43%), sputum (18.88%), high-quality cotton swabs (8.88%), inhalation swabs (7.77%), blood and wound cotton swabs (6.66%), pus (3.33%), bile (2.22%), and deep tissue (1.11%). Although more women than men were infected with *Candida* spp., the study found that those aged 51–60 years were most susceptible to *Candida* infections [20].

Another study reported Candida-associated UTIs as the most common nosocomial fungal infection worldwide [21]. Our study also identified *C. albicans* as the most common nosocomial fungal infection, but unlike the studies mentioned above, our study had a higher swab-positive rate. A culture and antifungal susceptibility study of *C. albicans* isolates from human blood cultures revealed that *C. albicans* was the most commonly found fungal species with a high infection rate in older adults (age ≥ 60 years) [22]. Therefore, an increase in infection prevention measures has been suggested in areas with a high population of older adults [22].

Contrary to this, our study showed the lowest infection rates in older adults (age ≥ 60 years) and higher positive rates among teenagers and those in their 20s. Furthermore, the specimens collected in our study consisted of swabs or urine, whereas the previous study used blood specimens. Thus, *C. albicans* isolated from blood cultures may present a higher infection rate in older adults and may suggest an association with weakened immunity. Other predisposing factors for the higher positive rates of Candida in older adults could include aging, diabetes, and malignancies [20].

Although several studies have investigated the positive rate of non-albicans *Candida* spp., none have exclusively examined *C. albicans* [23]. Therefore, more detailed research on the latter is warranted. Previous studies have shown a frequent occurrence of invasive *Candida* infections in the intensive care unit [24]. A limitation of our study is its inability to provide an association of *C. albicans* positivity with the patients’ symptoms or the presence of comorbidities.

Previous studies investigating STDs focused on understanding the epidemiology of viral infections (*i.e.*, HIV, hepatitis). However, epidemiological studies on fungi are very few. The recent clinical introduction of molecular biological testing and better testing capacity makes it possible to detect viruses and fungi that were previously difficult to culture or were time-consuming to detect. Future studies comparing the frequency and antifungal resistance between *C. albicans* and non-albicans strains are required.

## Figures and Tables

**Table 1:** Distribution of samples according to sex, age, and *C. albicans* positivity.

	Total (*n* = 59,381)		Male participants (*n* = 45,833)		Female participants (*n* = 13,548)	
Outcome of polymerase chain reaction test for *C. albicans*.						
Positive, n (%)	1,941	(3.2)	381	(0.8)	1,560	(11.5)
Negative, n (%)	57,440	(96.8)	45,452	(99.2)	11,988	(88.5)
Age, years (%)						
≤ 19	76	(7.4)	8	(1.1)	68	(22.7)
20–29	562	(3.5)	93	(0.7)	469	(16.9)
30–39	501	(3.2)	110	(0.8)	391	(14.0)
40–49	411	(3.7)	67	(0.8)	344	(12.2)
50–59	219	(2.5)	45	(0.7)	174	(6.7)
60–69	99	(2.1)	33	(1.0)	66	(4.5)
≥ 70	73	(3.0)	25	(1.6)	48	(5.7)
Average age, years	38.3		40.9		37.6	
*p*-value			0.0008		0.2968	
